# Separation of Respiratory Influences from the Tachogram: A Methodological Evaluation

**DOI:** 10.1371/journal.pone.0101713

**Published:** 2014-07-08

**Authors:** Devy Widjaja, Alexander Caicedo, Elke Vlemincx, Ilse Van Diest, Sabine Van Huffel

**Affiliations:** 1 Department of Electrical Engineering - STADIUS Center for Dynamical Systems, Signal Processing and Data Analytics, KU Leuven, Leuven, Belgium; 2 Medical Information Technologies Department, iMinds, Leuven, Belgium; 3 Faculty of Psychology and Educational Sciences - Health Psychology, KU Leuven, Leuven, Belgium; Charité - Universitätsmedizin Berlin, Germany

## Abstract

The variability of the heart rate (HRV) is widely studied as it contains information about the activity of the autonomic nervous system (ANS). However, HRV is influenced by breathing, independently of ANS activity. It is therefore important to include respiratory information in HRV analyses in order to correctly interpret the results. In this paper, we propose to record respiratory activity and use this information to separate the tachogram in two components: one which is related to breathing and one which contains all heart rate variations that are unrelated to respiration. Several algorithms to achieve this have been suggested in the literature, but no comparison between the methods has been performed yet. In this paper, we conduct two studies to evaluate the methods' performances to accurately decompose the tachogram in two components and to assess the robustness of the algorithms. The results show that orthogonal subspace projection and an ARMAX model yield the best performances over the two comparison studies. In addition, a real-life example of stress classification is presented to demonstrate that this approach to separate respiratory information in HRV studies can reveal changes in the heart rate variations that are otherwise masked by differing respiratory patterns.

## Introduction

The rate at which our heart beats, is a dynamical process enabling adaptive changes according to the demands of our body. These variations in heart rate are widely studied in so-called heart rate variability (HRV) analyses, as they contain much information about the activity of our autonomic nervous system (ANS). Furthermore, only the electrocardiogram (ECG) is required, making it a simple, noninvasive and popular tool. From the tachogram, the signal that represents the duration between consecutive heart beats, several HRV measures are defined to characterize ANS activity [1]. These variations in heart rate arise from several processes, such as thermoregulation, hormones, arterial blood pressure, respiration, etc. One of the main short-term modulators of the heart rate is respiration. This phenomenon is called respiratory sinus arrhythmia (RSA) and comprises the rhythmic fluctuation of the heart rate at respiratory frequency [2]. The presence of RSA is believed to improve pulmonary gas exchange [3] and two major mechanisms have been identified in the generation of RSA: modulation of cardiac parasympathetic activity by the central respiratory center; and inhibition of vagal efferent activity during inspiration by the lung stretch-receptor reflex [4]. Therefore, RSA is used as an index of vagal outflow. Typically, the respiratory frequency lies in the high-frequency (HF) band (0.15–0.40 Hz) of HRV and most HF power originates from RSA, making it an often used measure of RSA. However, problems arise when the respiratory frequency is around 0.15 Hz or lower. Then, it is difficult to interpret the HF power. One of the solutions is to use dynamic HF bands, as proposed in [5]. Another point of discussion originates from the interpretation of RSA measures. Especially in psychophysiology this is a highly debated topic as it is shown that the magnitude of RSA, either defined in time or frequency domain, changes with respiratory rate and the depth of breathing (tidal volume), independently of parasympathetic activity [6], [7]. It is therefore questioned whether RSA is a true index of vagal outflow. Proposed solutions include alternative calculations of RSA [7], [8] or statistical correction for differing respiratory parameters using ANCOVA with respiratory frequency and tidal volume as covariates [9]. For both sources of discussion, no solution has been acknowledged so far, leading to confusion in HRV analyses and questioning its use in practice. It is, however, apparent that it is important to include information of respiration when interpretations of HRV studies are made [10]. While most current research is focused on this RSA problem, we aim to investigate variations in the heart rate which are unrelated to respiration. More specifically, we propose to separate respiratory influences from the tachogram and thus to obtain a respiratory component of the tachogram and a residual tachogram that only contains variations in the heart rate that are not related to respiration. The goals of this approach are twofold; on the one hand we aim to improve the interpretation of HF power by including respiratory information in the analysis. On the other hand, we also believe that this approach might reveal changes in the tachogram that are otherwise dominated by respiratory effects and bring new insights to light.

In the literature, a few methods have been proposed to perform this separation. In this paper, we aim to conduct an extensive methodological comparison of the proposed methods, such as adaptive filtering [11], independent component analysis [12], system identification [13], multiscale principal component analysis [14] and orthogonal subspace projection [15], to identify the best and most accurate method to perform this separation. An overview of these methods is given in the next section. Then, the comparison is carried out using a simulation study that evaluates the correct decomposition of the tachogram. In addition, the robustness of each method will be evaluated. Note that in the literature also several physiology-based mathematical models of cardiorespiratory interactions have been proposed that make it possible to conduct the separation, such as [16], [17]. However, these approaches will not be evaluated in this paper since we focus on data-driven approaches. To conclude the paper, we will demonstrate that this separation should be considered in future HRV analyses using a real-life example of classifying stress periods based on the residual tachogram. The results of this example were presented at the 2013 IEEE EMBS Conference [18] and the paper was a finalist in the Student Paper Competition. This application clearly demonstrates the positive impact of the separation approach. Finally, a discussion on the conducted comparison studies and the real-life example is provided, followed by a conclusion.

## Separation Algorithms

In this section, we will give a summary of the algorithms that have been proposed in the literature to separate respiratory influences from the tachogram. All algorithms are based on the estimation of the respiratory component of the tachogram (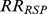) when the original tachogram (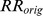) and a recorded respiratory signal (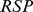) are given. The residual tachogram (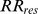) is then simply found by:
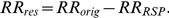
(1)


All signals are sampled at 4 Hz. MATLAB codes of all presented algorithms are available from the corresponding author upon request.

### 2.1 Adaptive Filtering

**Figure 1:**
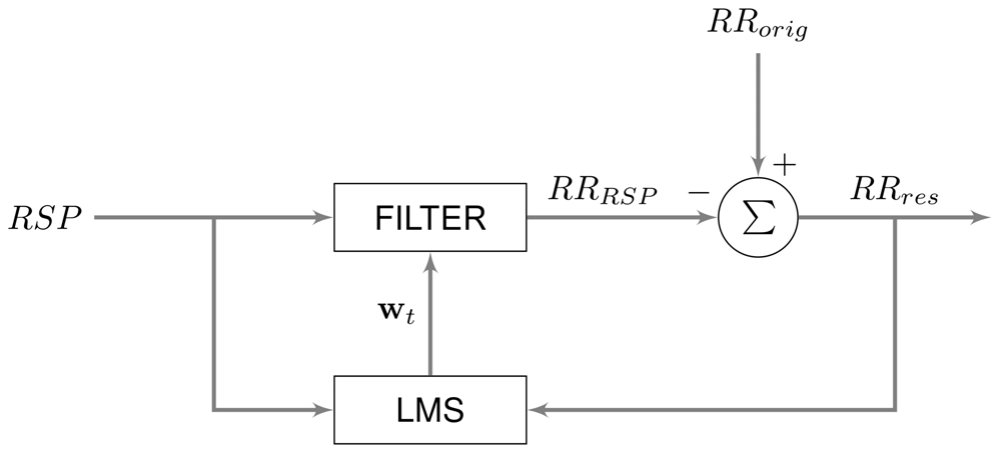
Scheme of the LMS adaptive filtering to separate respiratory influences from the tachogram.

The first method to estimate 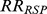 is based on adaptive filtering of the respiratory signal. Adaptive filtering for this application was first proposed by Bianchi et al. in [19] where they used a lattice adaptive filter. In 2008, least-mean squares (LMS) adaptive filtering was introduced to remove respiratory influences from tachogram and blood pressure to estimate the baroreflex sensitivity [11]. A few years later, the authors also applied it to estimate RSA or 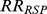 [20]. A scheme of the LMS algorithm is given in Fig. 1. Prior to application of the LMS algorithm, the respiratory signal and the tachogram are smoothed using a Savitsky-Golay filter of order 1 in windows of 5 samples, according to [11].

Next, each time sample 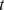 of 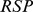 is filtered with a finite impulse response (FIR) filter to estimate 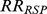, according to
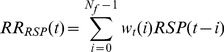
(2)with 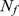 the number of adjustable filter coefficients 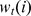. The LMS algorithm adapts the filter coefficients by minimizing the mean-squared error (MSE) between the tachogram and the estimate of the respiration component. A new set of filter coefficients 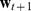 is then obtained iteratively by
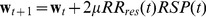
(3)where the parameter 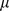 controls the stability and the rate of convergence.

The filter coefficients 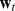 are initialized to zero. The entire signal is used to find the coefficients of the converging filter. These coefficients are then used as initial coefficients for the application of the LMS algorithm to the entire signal.

The appropriate selection of 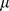 is important in order for the filter to work properly. The following constraint ensures convergence:
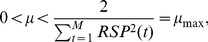
(4)with 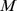 the number of samples in 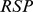.

In [11], a filter length of 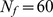 and convergence parameter 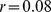 (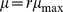) is chosen for a sampling frequency of 2 Hz. For this case, where a sampling frequency of 4 Hz is used, the parameters are set to 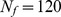 and 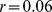.

More details on LMS adaptive filtering to estimate 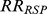 can be found in [11], [20].

### 2.2 Independent Component Analysis

Independent component analysis (ICA) is widely used to decompose observed data into its independent sources. ICA was proposed by Tiinanen *et al.* to separate respiratory influences from the tachogram [12].

Consider the observation vectors 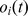 and unknown sources 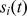, with 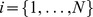 and 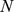 the number of sensors, and 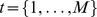 and 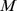 the number of samples in each observation vector, then we can write:
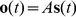
(5)with 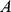 the mixing matrix. For this purpose, the respiratory signal and tachogram are the observation vectors:
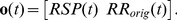
(6)


The expected independent sources are the respiratory component of the tachogram and the residual component:
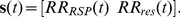
(7)


Note that the estimated sources are normalized, further annotated with subscript 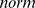. Therefore, scaling is needed to process the obtained signals in the correct units. A linear regression model is used to determine the scaling factors 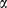 and 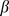:
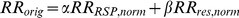
(8)

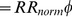
(9)with 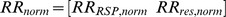 and 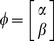.

Equation (9) can be solved via 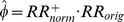 with 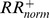 a pseudoinverse.

The FastICA algorithm was used to obtain the sources and mixing matrix [21]. This approach is explained in more detail in [12].

### 2.3 ARMAX Model

An autoregressive moving average with exogenous inputs (ARMAX) model was recently proposed to estimate 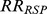 [13]. An ARMAX model estimates the output of the system as a linear combination of previous inputs, outputs and errors. In general, we can write 
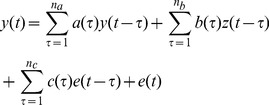
(10)with 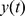, 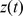 and 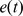 respectively the outputs, inputs and errors at time 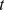; 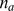, 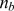, and 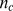, and 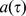, 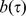 and 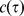 their respective model orders and predictor coefficients.

In this application, the model is simplified to
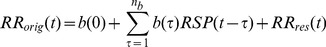
(11)with the original tachogram 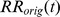 (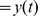) that is considered as a linear combination of 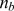 previous respiratory samples 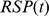 (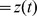) and a residual signal 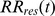 which contains all non-respiratory related processes. In [13], the chosen model order 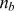 was not given. Preliminary experiments using values of 10, 20 and 50 for 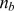 indicated that the predictor coefficients 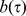 associated with delays larger than 8 samples are quasi zero. We therefore chose to use a delay (model order) 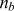 up to 3 seconds (12 samples) to ensure that all possible delayed effects of respiration on the tachogram are captured.

In matrix form, we can write
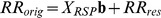
(12)with
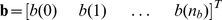
(13)

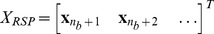
(14)

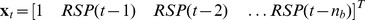
(15)and 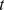 starting from sample 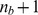.

The model coefficients 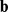 can be estimated through least-squares via
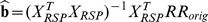
(16)


The respiratory component of the tachogram 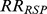 is computed as 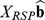. The residual signal 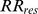 is then obtained by subtracting 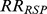 from the original tachogram. An elaborate description of this approach can be found in [13].

A very similar approach is described in [22], [23], where the tachogram is modeled as a sum of autoregressive processes of respiration, systolic blood pressure and other oscillations independent of respiration and blood pressure, in order to find reliable estimates of the baroreflex. When the blood pressure component is combined with the other oscillations, the same set-up as described above is obtained.

### 2.4 Multiscale Principal Component Analysis

Multiscale principal component analysis (MSPCA) “*combines the ability of PCA to decorrelate variables by extracting a linear relationship with that of wavelet analysis to extract deterministic features and approximately decorrelate autocorrelated measurements*” (p.1597) [24].

In MSPCA, the respiratory signal and original tachogram are first decomposed using wavelets, yielding detail coefficients 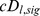 and approximation coefficients 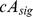, with 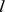 the decomposition level, and 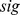 the signal (respiration 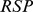 or tachogram 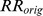). A Daubechies 4 wavelet is chosen as mother wavelet. The decomposition was performed up to level 5 under the assumption that no interaction between respiration and heart rate exists below frequencies of 0.0625 Hz, i.e. the upper bound of the frequencies contained in 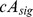 when a sampling frequency of 4 Hz is used. Note that the maximum level of decomposition also depends on the length of the data. In the next step, PCA is applied at each scale except for the approximation coefficients. If the first eigenvector explains over 90% of the variance in the data, the new wavelet coefficients are computed by projecting the coefficients onto the first eigenvector. Otherwise, the wavelet coefficients at that scale are set to 0. When we consider 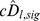 as the new wavelet coefficients, then the respiratory component of the tachogram can be constructed using 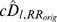. The obtained signal 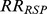 contains the component of the tachogram which is linearly related to the respiration. More details can be found in [14].

### 2.5 Orthogonal Subspace Projection

Orthogonal subspace projection (OSP) is used to decompose a signal in two independent components when a reference, in this case the respiratory signal 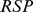, is given [15]. First, a subspace that is defined by the basis 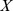 that represents respiratory activity is constructed. Then, the projection matrix 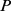, which projects the original tachogram onto the respiratory subspace, can be computed according to:
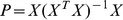
(17)


The resulting signal is the respiratory component 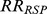 of the tachogram:
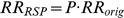
(18)


The respiratory basis 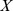 is constructed using the detail signals that are obtained from the wavelet decomposition of 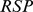 using Daubechies 4 wavelet up to level 5. The approximation signal is not included. In addition, a delay 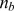 up to 3 seconds (12 samples, similar as in the ARMAX model) of each detail signal is included in the basis and the bias is estimated by addition of a column vector of ones in the basis 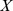. Consider the column vector 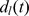, the detail signals of 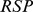, with 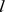 the level and 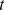 the samples 12 to 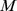, with 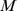 the total number of samples in 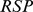, then the basis 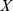 consists of 61 components:
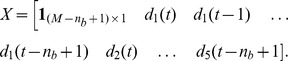
(19)


Note that the proposed OSP combines the strengths of the ARMAX model described in Sec. 2.3 and the multiscale approach in Sec. 2.4. More details about this approach are given in [15].

## Comparison Study

The performance of all proposed algorithms to decompose the original tachogram in two components is assessed using two comparison studies. The first study compares the ability of each method to accurately separate two signals using a simulation study. A second study is carried out to evaluate the internal stability or robustness of each algorithm.

### 3.1 Data

#### 3.1.1 Participants

The data used in this study originate from an experiment consisting of baseline recordings (resting sitting position) and conditions of induced worry and mindfulness, and were measured at the Faculty of Psychology and Educational Sciences of the KU Leuven (Leuven, Belgium) [25]. Recordings of 36 subjects (age 18–20) are used, of which one is randomly chosen as reference in the simulation study (cfr. Sec. 3.2, step 1). The remaining 35 participants constitute the test subjects. The experiment was approved by the Ethics Committees of the Faculty of Psychology and Educational Sciences and the Faculty of Medical Sciences of the KU Leuven and was in accordance with the Declaration of Helsinki. All participants were informed on the course of the experiment and provided written consent.

#### 3.1.2 Instrumentation

The electrocardiogram (ECG, sampling frequency 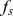 = 200 Hz) and respiration (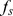 = 50 Hz) are measured by means of the LifeShirt System (Vivometrics Inc., Ventura, CA). Respiration was recorded using respiratory inductive plethysmography (RIP) around the ribcage (RC) and the abdomen (AB). The sum of RC and AB deflections is taken as an estimate of the tidal volume [26]. This volume will further be considered as the respiratory signal (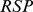).

#### 3.1.3 Preprocessing

Only the first 6 minutes of the baseline recordings are used in this study. The tachogram is constructed by detection of the R peaks in the ECG using the Pan-Tompkins algorithm [27]. All detections are manually inspected and corrected where needed. In order to prevent addition of colored noise in the HRV spectrum due to the low ECG sampling frequency, the R peak locations are enhanced using least squares parabolic interpolation on five samples surrounding the detected R peaks such that an accuracy of 1 ms is obtained [28]. Next, the respiratory signal and the tachogram are resampled at 4 Hz using cubic spline interpolation. In addition, the respiratory signal is filtered with a high-pass filter with a cut-off frequency of 0.05 Hz in order to remove baseline wander.

All processing steps of the data are performed in MATLAB R2012a (MathWorks, Natick, MA).

### 3.2 Simulation Study

#### 3.2.1 Experimental Setup

The first experiment is designed to evaluate how well each method can decompose a signal, i.e. a tachogram which is the sum of a respiratory component of the tachogram and a residual component, when the recorded respiratory signal is given. Since no ground truth for the two components is available, a simulation study is set up. In order to have realistic data, we chose to use real tachograms. Let algorithm 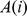 be one of the 5 proposed algorithms with 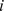 going from 1 to 5, let algorithms 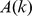 be all of the 5 proposed algorithms except for algorithm 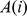, let 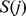 be the number of the subject with 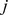 going from 1 to 35 and let 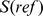 denote the reference subject with recorded signal 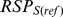 and 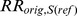. Then the experimental procedure is as follows:

1. Apply algorithm 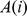 on the data of the reference subject. The respiratory component 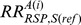 is taken as ground truth in the comparison later on.
2. On each of the 35 test subjects 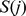 with data 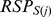 and 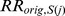, apply the same algorithm 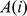 as in step 1 to remove the subjects' own respiratory component and obtain their residual components 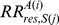.
3. Construct for each test subject 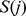 a new tachogram: 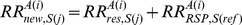.
4. Apply all algorithms 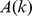 to decompose the tachogram in its two components. The inputs for the algorithms are 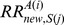 and 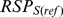. The obtained components are 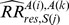 and 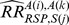.
5. Compare 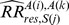 and 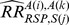 with 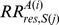 and 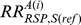, respectively using the evaluation measures described in the next section (Sec. 3.2.2).
6. Repeat steps 1 to 5 using each time a different algorithm 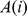 in step 1 and 2.

**Figure 2:**
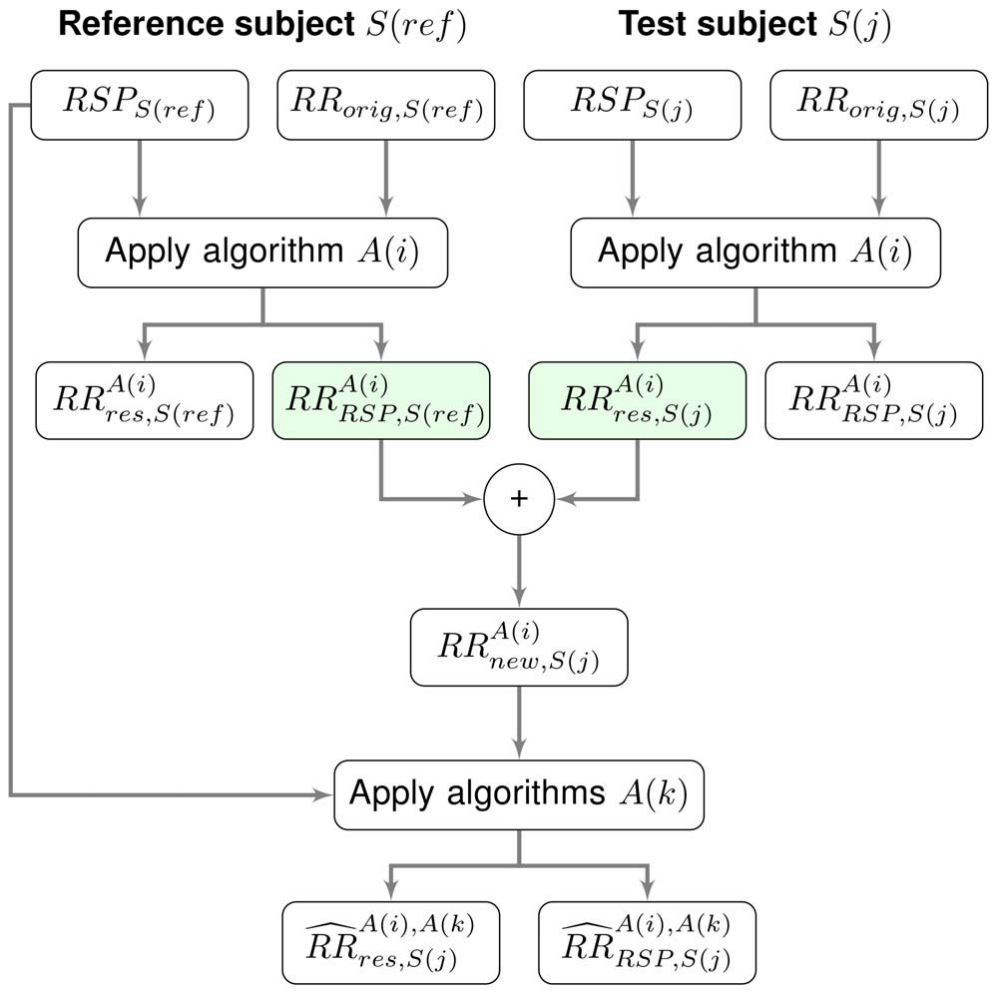
Block diagram of the simulation study.

A block diagram of steps 1 to 4 is shown in Fig. 2. This procedure can be seen as a cross-validation over the different algorithms to ensure that the evaluation of the performance is not biased by the use of the same algorithm in steps 1, 2 and 4.

#### 3.2.2 Evaluation Measures

The performance of all methods based on the simulation study is evaluated using two criteria of both the residual and respiratory component of the tachogram:

1. The normalized root-mean-squared error (NRMSE) of the time signals, which is defined as the root-mean-squared error divided by the range of the reference signal:
  - Residual component: 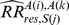 versus 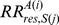

  - Respiratory component: 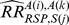 versus 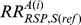

2. The squared errors (SE) of the low-frequency (LF: 0.04–0.15 Hz) and high-frequency (HF: 0.15–0.40 Hz) power:
  - Residual component: 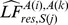 versus 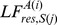, and 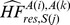 versus 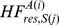

  - Respiratory component: 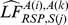 versus 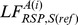, and 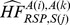 versus 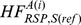

The spectra are computed via Welch's method, using a 1024 point fast Fourier transform, a periodic Hamming window of a length such that eight equal sections of the tachogram are obtained, each with an overlap of 50%.

#### 3.2.3 Results

**Figure 3:**
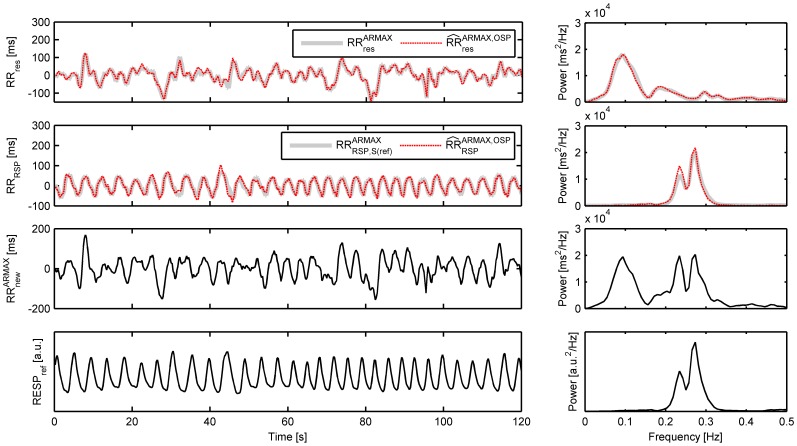
Example of the simulation study of a typical subject using the ARMAX model in step 1 and 2. Separation of the tachogram is performed using OSP. The signals are shown in both time (only 120 s are shown here) and frequency domain. From top to bottom: 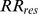, 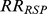, 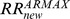, 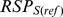. In dashed black, the estimated components using OSP are displayed.

Fig. 3 shows an example of the simulation study of a typical subject. The ARMAX model (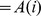) was used in step 1 and 2, and separation of the tachogram was performed using OSP (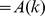). Both in time and in frequency domain, the resemblance between 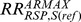 and 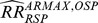, and 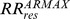 and 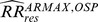 is high.

**Figure 4:**
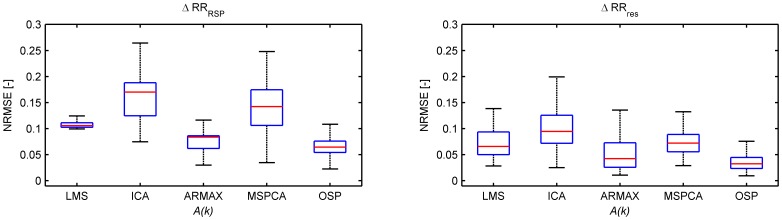
Boxplots of the normalized root-mean-squared errors (NRMSE) between 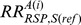 and 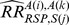 (left), and between 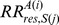 and 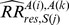 (right) obtained using the simulation study. Outliers are not shown.

**Figure 5:**
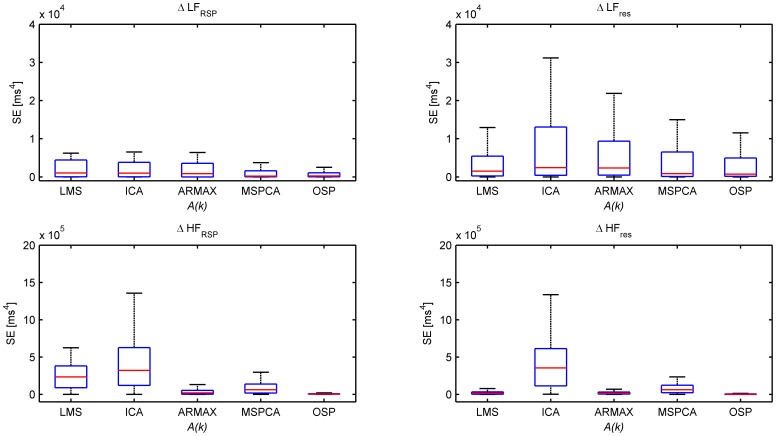
Boxplots of the squared errors (SE) in LF power (top) and HF power (bottom) between 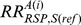 and 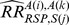 (left), and between 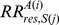 and 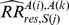 (right) obtained using the simulation study. Outliers are not shown.

The overall performance of all algorithms using the simulation study is shown in Figs. 4 and 5. Fig. 4 displays the NRMSE between the reference and the obtained signals. Both in the residual and in the respiratory component of the tachogram, the resulting signals are closest related to the reference signals using orthogonal subspace projection. The ARMAX model, LMS adaptive filter and MSPCA have an average performance while ICA does not show a good correspondence. These conclusions are confirmed when LF and HF power are compared, as in Fig. 5. Based on these 4 spectral indices and the NRMSE evaluation measures, OSP had the best overall performance in this simulation study.

When the results are analyzed in more detail, it appears that 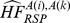 is mostly underestimated and 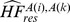 overestimated. Only OSP has a Gaussian distribution of errors around zero. This finding suggests that, except for OSP, the other algorithms might still contain respiratory influences in the residual tachogram. In 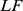, no consistent over- or underestimation is found.

In order to test the dependency of the results on the chosen reference subject, the analysis is repeated using other randomly chosen subjects as reference. These simulation studies yield similar results, and lead to the same conclusions.

### 3.3 Stability Study

#### 3.3.1 Experimental Setup

The goal of the second experiment is to assess how sensitive the methods are to the used data lengths by applying the algorithms on several window lengths. Let 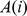 be one of the 5 algorithms used for the separation, and let 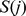 denote the number of the subject with 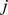 going from 1 to 36, then the following procedure is performed:

1. Apply each algorithm 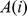 on the 6 minutes data 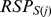 and 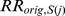 of every subject 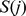. The obtained separated components of the tachogram are the reference for the following steps and denoted as 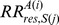 and 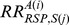.
2. Divide each tachogram 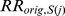 and respiratory signal 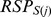 in 3 segments of 2 minutes and apply each algorithm 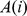 on every segment 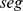, yielding 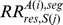 and 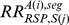.
3. Concatenate the three 2-minute segments:
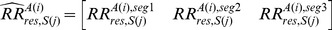


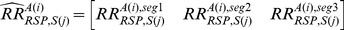

4. Compare for each algorithm 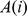 the acquired 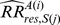 and 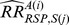 with the references 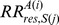 and 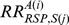 obtained in step 1 using the evaluation measures described in the next section (Sec. 3.3.2).

**Figure 6:**
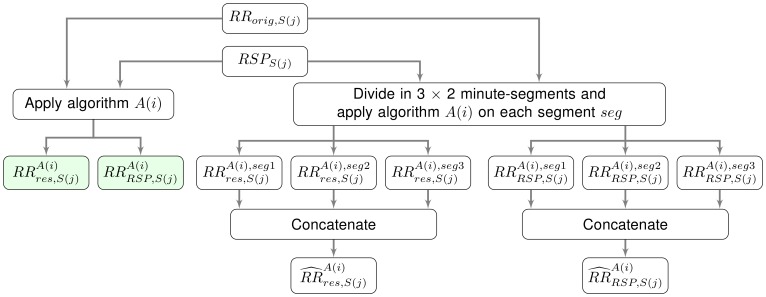
Block diagram of the stability study.

In the ideal case, concatenation of the three 2-minute segments yields the same signals as when the full 6 minutes are used. A block diagram of the stability study is given in Fig. 6.

#### 3.3.2 Evaluation Measures

Similarly as in Sec. 3.2.2, the performance of all separation methods is evaluated by the NRMSE of the time signals and the SE of the LF and HF powers. The stability of each algorithm is assessed by comparing 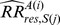 with 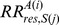, and 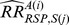 with 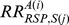.

#### 3.3.3 Results

**Figure 7:**
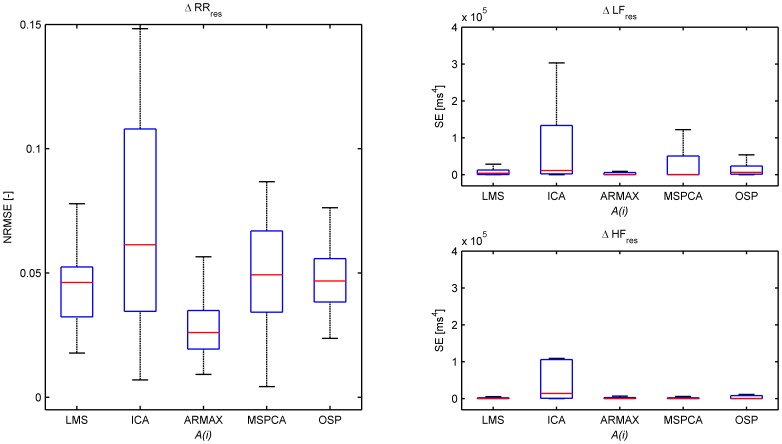
Boxplots of the normalized root mean-squared errors (NRMSE) (left), and the squared errors (SE) (right) in LF power (top) and HF power (bottom) between 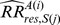 and 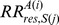 obtained using the stability study. Outliers are not shown.

**Figure 8:**
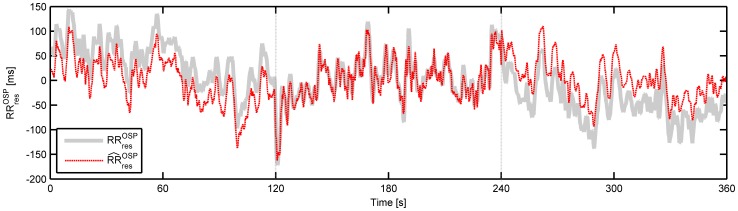
Example of stability study using orthogonal subspace projection. The residual signal of the full 6-minute period (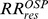, thick grey) and the three 2-minute segments (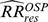, dashed black) are shown.

This second comparison study comprises a stability evaluation of each algorithm by changing the analyzed window size. This comparison is not designed to see how well the methods succeed in separating the tachogram in two components, but is set up to test the robustness of each method. The full window of 6 minutes is taken as reference while 2 minute windows are evaluated. Fig. 7 presents the NRMSE and SE of the LF and HF power of the residual tachogram. Comparisons using the respiratory component yield similar results and are not shown here. Based on the NRMSE, ICA shows a low stability. ARMAX has the lowest NRMSE, and surprisingly the performances of LMS, OSP and MSPCA are only average. Close inspection of the time signals, however, indicate that the low performance of OSP is due to an almost constant bias that is not captured in the smaller windows, as shown in Fig. 8. The very low frequency modulations cause a large NRMSE, and are in fact of no importance in most HRV studies as we are only interested in the dynamics in the LF and HF band. It is therefore more interesting to look at the performance based on the LF and HF power. These results indicate that LMS and ARMAX are least sensitive to window sizes when LF and HF power are compared. OSP shows only a moderate performance.

## Real-life Example: Stress Classification

The importance of conducting the separation of respiratory influences from the tachogram is demonstrated using the application of stress classification, which was also presented at the 2013 IEEE EMBS Conference [18]. We aim to show that the classification is enhanced using the residual tachogram instead of the original tachogram.

### 4.1 Data Aquisition and Preprocessing

The data were measured at the Faculty of Psychology and Educational Sciences of the KU Leuven (Leuven, Belgium) [29], [30]. The ECG (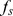 = 200 Hz) and respiration (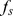 = 50 Hz) were recorded using the LifeShirt System (Vivometrics Inc., Ventura, CA).

The participants were instructed to perform, among others, twice a task that was designed to induce mental stress using arithmetic equations. Before, in between and after each task, there was a resting period. Each task had a duration of 6 minutes. For this real-life example, two randomly chosen resting periods and the two mental stress tasks (MT1 and MT2) of 40 students (age: 18–22 years) are used. The experiment was approved by the Ethics Committees of the Faculty of Psychology and Educational Sciences and the Faculty of Medical Sciences of the KU Leuven and was in accordance with the Declaration of Helsinki. All participants were informed on the course of the experiment and provided written consent.

The same preprocessing steps of the ECG and respiratory signal as described in Sec. 3.1.3 are applied. In order to increase the number of signals in the dataset, each period of 6 minutes is divided in segments of two minutes, with one minute overlap. This procedure results in 10 segments of rest and 10 segments of stress for each subject.

### 4.2 Classifier Design

A least squares support vector machines (LS-SVM) classifier is trained using a radial basis function (RBF) kernel and 5-fold cross-validation [31]. The data of 32 randomly chosen students compose the training set. The performance of the classifier is tested on the data of the remaining 8 subjects. This setup results in subject-independent classifiers.

The features used to classify the data segments are spectral indices of the original tachogram 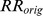, the residual tachogram 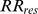 and the respiratory component of the tachogram 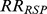, obtained using OSP. Both LF and HF power are computed. In addition, 
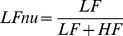
 (normalized units), 
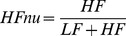
, the ratio 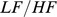 and the power in the total frequency band 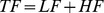 are considered. These spectral indices are computed for three classifiers using 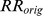, 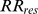 and 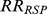 separately. In order to make a fair comparison with the respiratory and residual tachograms, that are obtained using additional respiratory information, a classifier that combines features of the recorded respiration with the information of the original tachogram is evaluated. The additional features derived from the respiratory signal (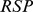) are 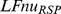, 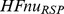 and 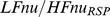. Note that the normalized power is used as the respiratory signal is in arbitrary units.

### 4.3 Results

**Figure 9:**
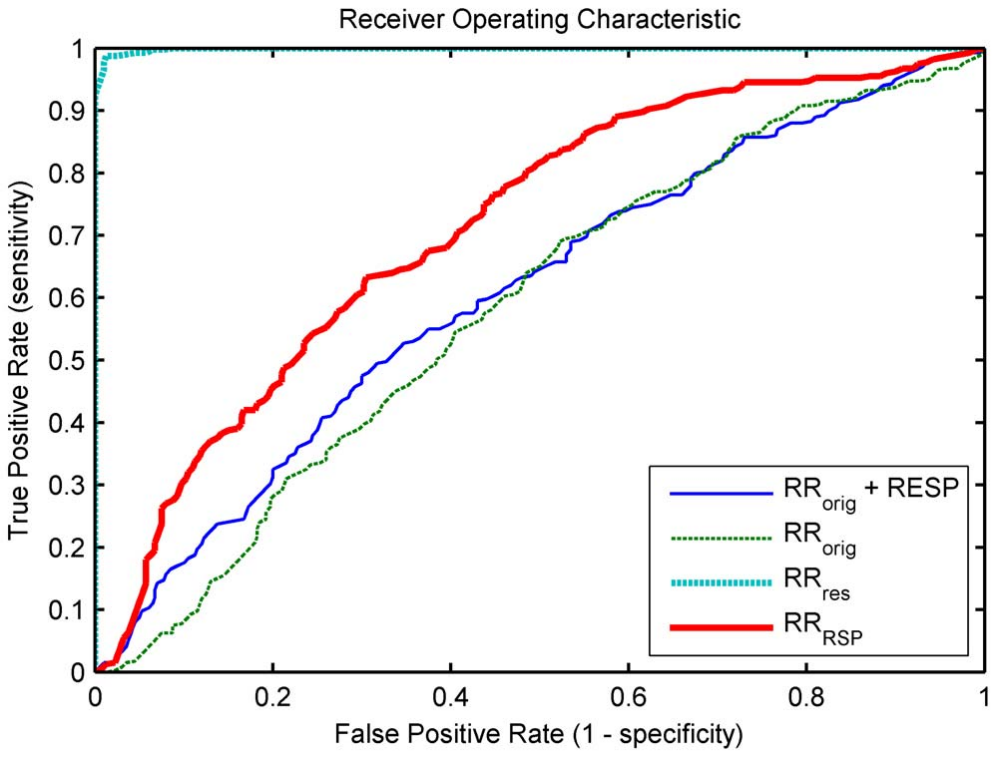
Mean ROC of all classifiers.

The performance of each classifier is assessed by averaging over 5-fold classification using different training and test sets. Consider stress as the positive class and rest as the negative one, then the mean ROC of each classifier is shown in Fig. 9. Based on 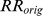, the classification in rest and stress has an accuracy of merely 57.13%. Inclusion of information derived from the respiratory signal does not improve the results (accuracy  = 57.88%). A slightly better accuracy of 66% is obtained using only 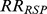. An apparent improvement is found when 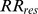 is used, yielding an almost perfect classification (accuracy  = 97.88%). A more elaborate analysis of the results can be found in [18].

## Discussion

In this study, the challenge of respiratory influences in the tachogram is handled by the approach to separate respiratory influences in heart rate variations from non-respiration related changes. In the literature, several methods have been proposed to deal with this problem, but no comparison between the algorithms has been performed. Therefore an extensive comparison using a simulation study and a stability study has been set up. All data-driven algorithms, up to our knowledge, that appeared in the literature to conduct this cardiorespiratory separation were evaluated. Note that the algorithms were implemented as they appeared in the literature, and thus no further optimisation of parameters was conducted in this comparison study. In addition, the importance of such approach was demonstrated in a real-life example.

### 5.1 Comparison Study

The first comparison of algorithms is carried out in a simulation study which was designed to examine which method performs best given two realistic signals of the residual and respiratory component of the tachogram, which are, moreover, also known. We did not use purely simulated signals as they would only provide us a theoretical evaluation of the best method. Moreover, the generation of simulated data requires several preassumptions, such as the extent to which the spectra of the respiratory and residual tachogram overlap, and their relative contributions to the original tachogram. In order to obtain two realistic signals, each of the proposed algorithms was used to estimate a residual tachogram and a respiratory component which has no connection with the residual tachogram as data of another subject were used, and the performance of the remaining algorithms is then evaluated. This cross-validation between the methods ensures a fair comparison and is needed to be implemented as no ground truth of the separated signals is available. The second comparison study is designed to evaluate the robustness of each algorithm as it is important that the algorithm yields similar time signals when a window of 6 minutes is considered than when windows of 2 minutes are used. Without doubt, a good performance in the simulation study is of prime importance; without a correct separation, a good stability is of no use. Conversely, a good separation without a high performance in the stability study should not discard the use of the algorithm involved, but displays the limitations that should be taken into account.

The simulation study revealed that LMS adaptive filtering has an average performance. Closer inspection of the LMS approach reveals that the use of the Savitsky-Golay filter is an important factor in the performance; when the signals are not smoothed, the performance of adaptive filtering decreases significantly. We should therefore consider including this filter in the other algorithms to further improve the results. Note that the use of a higher order Savitsky-Golay filter will result in limited smoothing, thereby abolishing the beneficial effect of the filter. Although it was expected that adaptive filtering would be least affected by different window sizes, the stability study showed that this method is not as robust as hypothesized.

Both comparison studies indicated that ICA is not a suitable method to separate respiratory influences from the tachogram. Although it was shown in [12] that ICA is a good method to do so, and it does not change the power content, this study shows the inverse. In [12], the simulation study was conducted using LMS adaptive filtering in step 1 and 2, followed by ICA. Our results show that spectral features, especially the HF power, of 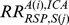, are largely underestimated using ICA. A possible explanation of the low performance can be found in the presupposition of statistical independency between respiration and heart rate, which can not be assumed. In addition, the stability study showed very low robustness.

MSPCA also shows an average performance. The LF power of the respiratory signal is generally overestimated, while this feature is mostly underestimated in the other algorithms. The main advantage of this method is that several scales are considered, but some arbitrarily chosen parameters largely influence its performance, e.g. the cut-off variance to define when there is a clear relation.

Another method included in our study is the ARMAX model. Generally, this method shows a good performance in the comparison studies. However, the performance can possibly be improved by optimizing the model order. This was fixed at 3 seconds (or 12 samples) to ensure that all possible delayed effects of respiration on the tachogram are taken into account in the model. Nonetheless even without optimization, a satisfactory performance is obtained. In [13] they use this approach to make a model using broadband spectrum. This broadband spectrum was realized using paced breathing. Because it is shown that paced breathing increases the tidal volume [32], they propose a scaling factor to normalize the model for spontaneous breathing. We did not include this procedure as no data were available. In addition, we aim to use this for already analyzed HRV studies, which do not have such broadband breathing spectrum.

Orthogonal subspace projection combines the advantages of MSPCA and ARMAX and also shows to have the best performance. In the simulation study, OSP was the only method of which the difference in spectral features had a distribution around zero. However, similarly as with the ARMAX model, an optimization of the model order might improve the results, as well as the choice of the mother wavelet and its order. The limitation of this method is clear in the stability study; although the spectral features are still reasonably estimated, NRMSE shows that there are discontinuities when the signal is cut in smaller parts. Future research should focus on improving the stability using overlapping windows and subspace tracking to reduce border discontinuities and bias differences, as suggested in [33].

From all the results, we can conclude that OSP and ARMAX are the most reliable methods to separate the tachogram in a respiratory component and a residual tachogram. OSP shows to have the best performance in the simulation study, but the moderate robustness limits its use for longer data. In that case, the ARMAX model should be used.

In future studies, several issues still need to be investigated such as the importance of the measured respiratory signal. In this comparison study, only estimates of the tidal volume are used. The sensitivity to the type of respiratory recording should be examined, as well as the importance of the recording quality. In addition, it should be investigated whether the separation would benefit from nonlinear approaches; in a first study, we only considered linear separation algorithms that were used in the literature. Consequently, possible nonlinear interactions are not taken into account now.

HRV and respiratory oscillations are closely related to blood pressure variations. Indeed, heart rate and blood pressure interact via the feedback (i.e. baroreflex path) and feedforward (i.e. Windkessel effect) mechanisms, and are both influenced by respiration. Hence, many studies have been conducted to model those signals and their interactions, as in [22], [23], [34], [35]. These models could also be used to estimate HRV unrelated to respiration if blood pressure recordings are available. Note however that the present study aimed at investigating the tachogram from which both direct and indirect (via blood pressure) influences of respiration are separated from other sources of HRV, without needing to explicitly define the blood pressure relation to HRV. In this way, HRV analyses can be conducted that are not influenced by the highly debated interaction with respiration. Evidently, one source of the residual tachogram is related to blood pressure and further research is needed to identify other sources of the residual tachogram.

### 5.2 Stress Classification

The importance of separation of the original tachogram was demonstrated using a real-life example of stress classification. The results show that classification using 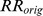, with and without additional respiratory information, is almost random. We hypothesized that the original tachogram contains HR modulations of respiration and non-respiration related variations, and that separation of both will lead to an increased performance when rest and stress are classified. This study confirms this; a slightly better performance is obtained when spectral features of 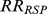 were used for the classification, but an almost perfect classification was obtained using the residual tachogram. These findings demonstrate that the original tachogram contains HR variations, unrelated to respiration, that seem to be very important to distinguish stress from rest, but these variations might be dominated by the strong respiratory influence on the heart rate. Although it has been observed that stress influences the respiratory pattern [14], [29], 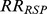 did not lead to the expected increase in performance.

An important advantage of the followed methodology is the subject-independent classification. In most cases, stress has been considered as a subject-dependent phenomenon and classification was performed in a subject-specific manner, as in [13].

In this application, OSP was chosen to separate respiratory influences from the tachogram as the data segments have a length of only two minutes. However, in a preliminary study, we also conducted the same stress classification using the ARMAX method, from which we can conclude that similar classification results can be obtained. Possibly, due to the quasi-constant breathing frequency during those two minutes, the strength of the wavelet decomposition in OSP did not need to be exploited, leading to comparable results. Note that we did not conduct stress classification using the other algorithms as they did not provide a reliable separation of respiratory influences from the tachogram. Even if they would obtain good classification results, we would not know whether respiratory or other influences are removed or not, which would lead to problematic interpretations of the results.

A more elaborate evaluation of stress classification using OSP can be found in [18]. However, from these observations we can deduce that the influence of respiration on HRV might not only complicate interpretations regarding ANS activity, it might actually disguise valuable information in the tachogram on efferent ANS activity. These results motivate the conducted comparison study and demonstrate the importance of the separation in stress classification. However, further studies should confirm the added value of the separation of the tachogram in other cardiorespiratory applications such as analyses of coupling; i.e. this approach might complement analyses of cardiorespiratory coupling by means of phase synchronization between the heart rate and respiration [36]. In addition, future research is needed to determine the physiological meaning of the residual tachogram.

## Conclusion

Cardiorespiratory interactions, such as RSA, are widely studied as they contain information about our autonomic nervous system and cardiac vagal outflow. Whereas most research focuses on finding the correct interpretation of RSA, this research aimed at separating variations in the heart rate that are related to respiration from HR variations that are not related to respiration. Five algorithms have been proposed in the literature to conduct this separation and are now extensively compared to determine which method is the best to accurately decompose the tachogram in two components. In addition, the robustness of the algorithms is evaluated. The results show that the algorithms based on orthogonal subspace projection and on the ARMAX model yield the best performances, and are therefore advised to be used in future studies.

The importance of separating respiratory influences from the tachogram is demonstrated in the application of stress classification where we showed that the HR variations unrelated to respiration yield almost perfect classification whereas the use of the original tachogram fails. These results prove that the separation of the tachogram can be a very useful tool to investigate changes in the heart rate that are otherwise masked by the dominant effect of respiration.
